# Modulatory Effect of Medicinal Plants and Their Active Constituents on ATP-Sensitive Potassium Channels (K_ATP_) in Diabetes

**DOI:** 10.3390/ph16040523

**Published:** 2023-03-31

**Authors:** Lina T. Al Kury

**Affiliations:** Department of Health Sciences, College of Natural and Health Sciences, Zayed University, Abu Dhabi 144534, United Arab Emirates; lina.alkury@zu.ac.ae

**Keywords:** diabetes mellitus, medicinal plants, pancreatic ATP-sensitive potassium channels, insulin secretion

## Abstract

Hyperglycemia, which is a chronic metabolic condition caused by either a defect in insulin secretion or insulin resistance, is a hallmark of diabetes mellitus (DM). Sustained hyperglycemia leads to the onset and development of many health complications. Despite the number of available antidiabetic medications on the market, there is still a need for novel treatment agents with increased efficacy and fewer adverse effects. Many medicinal plants offer a rich supply of bioactive compounds that have remarkable pharmacological effects with less toxicity and side effects. According to published evidence, natural antidiabetic substances influence pancreatic β-cell development and proliferation, inhibit pancreatic β-cell death, and directly increase insulin output. Pancreatic ATP-sensitive potassium channels play an essential role in coupling glucose metabolism to the secretion of insulin. Although much of the literature is available on the antidiabetic effects of medicinal plants, very limited studies discuss their direct action on pancreatic K_ATP_. The aim of this review is to focus on the modulatory effects of antidiabetic medicinal plants and their active constituents on pancreatic K_ATP_. The K_ATP_ channel should be regarded as a key therapeutic milestone in the treatment of diabetes. Therefore, continuous research into the interaction of medicinal plants with the K_ATP_ channel is crucial.

## 1. Introduction

Hyperglycemia, which is a chronic condition caused by either insulin secretion problems or insulin resistance, is a hallmark of diabetes mellitus (DM). Numerous complications, such as the production of reactive oxygen species (ROS), which cause the oxidation of lipids and membrane damage, are brought on and developed by prolonged hyperglycemia. Additionally, the excessive non-enzymatic glycation of proteins and the production of advanced glycation end products (AGE) are formed [1]. As a result of glycation changes, the pathophysiology of diabetes can become worse. Diabetes management continues to be a major issue on a global scale, and a cure has not yet been found. According to the World Health Organization (WHO), currently, about 350 million individuals in the world have diabetes [2].

Despite the fact that there are several well-known synthetic antidiabetic medications, their ineffectiveness and undesirable side effects have increased interest in finding novel and alternative antidiabetic treatments. Because of their capacity to boost insulin production, reduce glucose absorption via the intestine, and alter insulin sensitivity, a number of medicinal plants have recently been found to be beneficial for antidiabetic treatment [3,4]. Flavonoids, terpenoids, alkaloids, carotenoids, and other secondary metabolites from medicinal herbs have been documented for their safe action in diabetes [3,5]. Over 800 plants have been claimed to possess antidiabetic properties with no known adverse effects and low toxicity in comparison to synthesized drugs [6]. However, the search for new antihyperglycemic drugs from natural plants which have safe effects is still desirable.

## 2. Therapeutic Options and Their Limitations

Currently, there are five classes of oral hypoglycemic drugs: each work differently to treat the underlying causes of diabetes. The mechanisms by which a hypoglycemic effect is achieved include (1) the stimulation of insulin production (sulfonylureas and meglitinides), (2) stimulation of peripheral glucose absorption (thiazolidinediones and biguanides), (3) the delay of carbohydrate absorption from the intestine (alpha-glucosidase), and (4) a reduction in hepatic gluconeogenesis (biguanides). Lifestyle modifications combined with the use of hypoglycemic agents are important to accomplish long-term metabolic control in diabetes [7]. Despite this, up to date, complete recovery from diabetes has not been reported, and drug treatment does not always prevent late-stage diabetic problems [8].

Although the early effects of oral hypoglycemic medications may be helpful, they may also gradually lose their potency in a significant majority of diabetic patients. Currently, available medications have a number of negative effects. For instance, sulfonylurea can lead to weight gain and hyperinsulinemia. Meglitinides have a similar mechanism of action to sulfonylureas [9], but they have a lower risk of weight gain and hypoglycemia episodes, making them a better alternative for patients who experience these adverse effects [10]. Other side effects include fatigue and lactic acidosis (biguanides), flatulence and diarrhea (alpha-glucosidase inhibitor), and an elevated LDL-cholesterol level (thiazolidinediones) [11].

## 3. Role of K_ATP_ Channels in Diabetes

Adenosine triphosphate-sensitive potassium (K_ATP_) channels of pancreatic β-cells are crucial for glucose metabolism because they control insulin production in response to an increase in ATP concentration [12]. K_ATP_ channels have the general purpose of coupling membrane potential and potassium (K^+^) conductance to the metabolic state of the cell via detecting the intracellular ATP content. It is generally known that glucose stimulates the release of insulin from β-cells through glycolysis and mitochondrial oxidation, which raises the intracellular ATP/ADP ratio. As a result, K_ATP_ channels close, resulting in membrane depolarization. This then causes the plasma membrane’s voltage-gated L-type Ca^2+^ channels to open, Ca^2+^ to enter, and the exocytosis of insulin-secreting granules to be activated [13].

The β-cell K_ATP_ channel is a multimeric complex made up of two different types of subunits: a pore-forming component, Kir6.2, and a regulatory subunit, the sulfonylurea receptor SUR1 [12,14]. For K_ATP_ channels to operate, both subunits must be present. The Kir6.2 location is where ATP mediates channel blockage, while the SUR1 nucleotide-binding domain is where MgADP activates the channel. K_ATP_ channels are thought to be interesting targets for the development of medications for the treatment of diabetes because they have a rich and diversified pharmacology with many substances acting as particular inhibitors or activators. Effective insulin secretagogues, such as glibenclamide and tolbutamide, bypass cell metabolism and directly block K_ATP_ channels to cause insulin release [15,16]. In this review, we focus on the interaction of medicinal plants and their active constituents with the K_ATP_ channel. The K_ATP_ channel should be regarded as a key therapeutic milestone in the treatment of diabetes. Therefore, understanding the mechanism behind the interaction of medicinal plants with the K_ATP_ channel can facilitate drug development for the treatment of diabetes.

## 4. Effect of Medicinal Plants and Their Active Constituents on K_ATP_ Channel

### 4.1. Lupinus mutabilis

*Lupinus mutabilis* is a native legume of South America that belongs to the Fabaceae family. The plant is often referred to as Tarwi or Andean lupin. Previous in vitro and in vivo studies provided evidence that *Lupinus* species have a hypoglycemic activity which was attributed to the presence of the protein γ-conglutin as well as quinolizidine alkaloids [17,18]. To date, more than 20 distinct quinolizidine alkaloids have been identified in the *L. mutabilis* alkaloid fraction [19].

**Figure 1:**
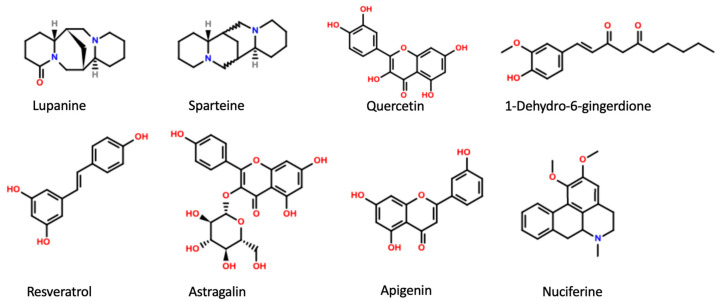
Main active plant constituents with inhibitory effect on K_ATP_ channels.

Earlier studies examined the effect of raw *L. mutabilis* on blood sugar and insulin levels in normal and dysglycemic human subjects. The consumption of *L. mutabilis* by hyperglycemic patients resulted in a considerable decrease in blood glucose levels, while consumption of the same doses by healthy adults did not cause a significant alteration in blood glucose and insulin levels. Interestingly, the effects of *L. mutabilis* were stronger in people with greater basal glucose levels. In addition, treatment with *L. mutabilis* reduced insulin resistance in patients with diabetes [20]. Cooked *L. mutabilis* seeds have also been shown to lower glycemia in people with type 2 diabetes, according to clinical research. This action is likely due to the alkaloid content of *L. mutabilis* [21]. Wiedemann et al. looked at the effect of lupanine (Figure 1), one of the main quinolizidine alkaloids found in *Lupinus mutabilis* seeds, on a clonal rat-derived β-cell line (INS-1E) and on rats pre-treated with streptozotocin (STZ). The potentiating effect of lupanine began to manifest in L-arginine-treated islets at a glucose concentration of 8 mmol/L. Lupanine also increased the expression of the Ins-1 gene. Electrophysiological studies have shown that lupanine directly and dose-dependently blocked K_ATP_ channels. An analysis of the stimulus-secretion coupling revealed that membrane depolarization and the increase in the frequency of Ca^2+^ action potentials (APs) were linked to the potentiating impact on secretion. In diabetic rats pretreated with STZ, lupanine reduced plasma glucose levels and boosted glycemic control. Lupanine dramatically increased insulin secretion in the presence of 15 mmol/L glucose. Although orally administered lupanine did not cause hypoglycemia, it improved glycemic control in STZ-diabetic rats [22]. The finding that the effect of lupanine depends on the glycemic status of the subject is in agreement with the human studies mentioned above [20]. The lupin alkaloid sparteine has also been demonstrated to block K_ATP_ channels in mouse β cells and in the insulin-secreting cell line (HIT-T15). When compared to lupanine, sparteine’s inhibitory impact was far more obvious and strong enough to cause insulin secretion in unstimulated islets at micromolar doses [23,24].

### 4.2. Belamcanda chinensis

*Belamcanda chinensis* is a member of the Iridaceae family. The dried rhizome of *B. chinensis* was traditionally utilized to treat throat disease and inflammation in Chinese medicine [25]. Several flavonoids were identified in the leaves of the plant [26]. Wu et al. studied the effect of the aqueous extract of *B. chinensis* leaves on normal and STZ-induced diabetic rats. The *B. chinensis* extract (400, 800, 1600 mg/kg, p.o.) decreased oral glucose tolerance and fasting blood glucose levels in both healthy and diabetic rats. Additionally, it raised serum insulin levels in healthy rats. This effect was reversed in the presence of the K_ATP_ channel opener nicorandil and Ca^+2^ channel blocker nifedipine. The results indicate that the *B. chinensis* extract induces insulin release via the K_ATP_ channel-dependent pathway [27]. The results of a recent study conducted by the same group provided evidence that the extracts of *B. chinensis* leaves significantly decreased hyperglycemia, ameliorated insulin sensitivity, and prevented hepatic gluconeogenesis in KK-A^y^ mice [28].

### 4.3. Hyphaene thebaica

*Hyphaene thebaica* (Areceaeae), also known as doum, is a type of palm tree commonly found in Egypt, Sudan, and other countries in Africa. The fruit of *H. thebaica* is edible and rich in proteins, carbohydrates, lipids, iron, calcium, and other minerals. The fruit has a variety of phytochemical substances, such as flavonoids, hydroxy cinnamates, essential oils, and saponins. A phytochemical analysis of *H. thebaica* revealed the presence of more than ten different flavonoids, in addition to tannins, saponins, glycosides, and essential oils [29,30]. The antidiabetic potential of *H. thebaica* has been reported in earlier studies. In alloxan-induced diabetic rats, the water-soluble fraction of the fruit enhanced glucose and insulin tolerance and significantly decreased glycosylated hemoglobin levels [29]. In STZ-treated diabetic rats, the aqueous extracts of *H. thebaica* caused a significant reduction in blood glucose levels, improved the relative expression of insulin, and decreased the relative expression of inflammatory mediators TNF-α and TGF-β. According to histopathological studies, treatment with the aqueous extract successfully reversed the β-cell necrosis brought on by STZ and returned it to its normal form. The pancreatic K_ATP_ channel was identified as a target for flavonoids: the principal bioactive phytochemicals present in the aqueous extract of *H. thebaica* [31]. Although many flavonoids extracted from *H. thebaica* have been shown to increase glucose and insulin tolerance and reduce blood levels of glycosylated hemoglobin [29], their antidiabetic effects have not been fully elucidated. In addition, the data on the effects of flavonoids on pancreatic K_ATP_ are very scarce.

### 4.4. Eucalyptus citriodora

*Eucalyptus citriodora* (lemon-scented gum) belongs to the Myrtaceae family. This plant is known for its analgesic, anti-inflammatory, antioxidant, antimicrobial, and antipyretic activities [32,33,34]. According to earlier research, *E. citriodora* has characteristics that can be used in the treatment of diabetes. The phytochemicals in *E. citriodora* have a range of antidiabetic effects that are mediated by several mechanisms. It has been demonstrated that *E. citriodora* reduces hyperglycemia in alloxan-treated diabetic rats. The plant leaves contain betulinic acid and corosolic acid, which have been shown to have a hypoglycemic effect by promoting the translocation of GLUT-4 [35]. In BRIN-BD11 cells and isolated mouse pancreatic islets, *E. citriodora* leaves stimulated concentration-dependent insulin release from β-cells. Interestingly, this effect was inhibited by diazoxide, verapamil, and extracellular Ca^2+^ depletion, suggesting that K_ATP_ channels, L-type Ca^2+^ channels, and the Ca^2+^ influx were involved in the insulin secretory activities. Supporting this finding, the extract depolarized the plasma membrane of β-cells and caused an increase in intracellular Ca^2+^. In high-fat-fed diabetic rats, the oral application of *E. citriodora* for nine days enhanced glycemic control and β-cell activity. These effects were attributed to the presence of phytochemicals such as quercitrin, isoquercitrin, and rhodomyrtosone E [32]. In fact, the effect of quercetin (which exists mostly in its glycoside form quercitrin) on insulin release was reported earlier. Electrophysiological studies have shown that quercetin depolarized the β-cells membrane, stimulated an increase in intracellular Ca^2+^ concentration, and caused a 50% decrease in the current through K_ATP_ channels [36].

### 4.5. Leonurus sibiricus

*Leonurus sibiricus* is an herbaceous plant that belongs to the family Lamiaceae which was found in various locations in Asia and America. Many Asian nations have utilized *L. sibiricus* in traditional medicine to treat type 2 DM. The literature has several reports on *L. sibiricus’s* pharmacological properties. For instance, the plant has been demonstrated to display significant antioxidant as well as anti-inflammatory action in rats and humans [37].

Schmidt et al. studied the impact of the *L. sibiricus* plant extract on INS-1E insulinoma cell proliferation, electrophysiological characteristics, intracellular Ca^2+^ concentration, and insulin production. Both the aqueous and methanolic extracts from *L. sibiricuss* dramatically boosted the rat INS-1E cells’ ability to secrete insulin. The aqueous extract blocked K_ATP_, depolarized membranes, and raised intracellular Ca^2+^ concentrations when applied acutely. The electrophysiological effects were intriguingly similar to those of tolbutamide: the sulfonylurea antidiabetic agent. Additionally, the administration of the aqueous extract induced INS 1-E cell growth. The results of this investigation lend support to the use of *L. sibiricus* in conventional medicine for the treatment of DM [38]

The β-cells responses to flavonoid quercetin and its glycoside rutin, which are present in the *L. sibiricus* extract, were examined by Kittl et al. [36]. Rutin did not affect insulin secretion or the electrophysiological activity of rat INS-1 cells, despite the fact that these phytochemicals are known to possess insulinotropic characteristics in vivo and in vitro studies. The transitory K_ATP_ channel blockage and intracellular Ca^2+^ stimulation of quercetin, on the other hand, likely contributed to its ability to abruptly promote insulin secretion.

### 4.6. Portulaca oleracea

*Portulaca oleracea* is a member of the Portulacaceae family that is extensively found throughout temperate and tropical regions of the world. The plant has been reported to have many biological activities, such as hypoglycemic, antioxidant, and anti-inflammatory effects. Alkaloids, terpenoids, coumarins, and flavonoids have all been found in this plant [39].

The effect of the *P. oleracea* extract on insulin secretion in INS-1 pancreatic β-cells and its mechanism of action were studied by Park and Han. The results of this investigation demonstrated a dose-dependent rise in insulin production by INS-1 pancreatic β-cells in response to *P. oleracea* extract application. The co-administration of diazoxide or verapamil, along with the *P. oleracea* extract, blocked membrane depolarization and Ca^2+^ influx and, consequently, inhibited insulin secretion. The findings imply that the membrane depolarization and closing of K_ATP_ channels in INS-1 pancreatic cells may be related to the insulinotropic action of the *P. oleracea* extract. Supporting these results, treatment with *P. oleracea* extract, along with the depolarizing concentration of KCl, greatly enhanced insulin secretion in comparison to treatment with KCl alone [40]. It is important to mention that *P. oleracea* is rich in many phytochemicals, such as polyphenols, terpenoids, and alkaloids [39]. For example, the triterpenoid 3β-hidroxihop-22(29)ene (3-BHO) was found to regulate the Ca^2+^ influx through the K_ATP_ channel-dependent route in rat pancreatic islets [41]. Collectively, the insulin secretory effects of *P. oleracea extract* could be attributed to the presence of active phytochemicals such as flavonoids and triterpenoids.

### 4.7. Cichorium intybus

*Cichorium intybus*, commonly known as chicory, is a member of the Compositae family. It is widely used in India as a traditional treatment for diabetes mellitus. Pushparaj et al. tested the antidiabetic effect of this plant on STZ-induced diabetic rats [42]. The results of this study showed that the daily administration of the ethanolic extract of *C. intybus* (125 mg/kg) for 14 days attenuated serum glucose by 20%. A similar finding was observed in a more recent study where the administration of the ethanolic extract to STZ-induced diabetic rats lowered plasma glucose and improved their lipid profile. In addition, the extract had antioxidant properties and modulated an oxidative stress system in diabetic rats [43].

Resveratrol is one of the polyphenols that are present in large amounts in *C. intybus* [44]. Resveratrol is well-reported for its potent antidiabetic actions by reducing glucose levels, ameliorating the parameters of oxidative damage, and preserving β-cells [45]. A whole-cell voltage-clamp study on a mouse β-cell line revealed that resveratrol significantly inhibited the K_ATP_ current at a concentration of 3 μmol/l and stimulated insulin secretion by causing membrane depolarization [46]. Supporting this finding, Hambrock et al. demonstrated that resveratrol has the ability to bind to SUR, the regulatory subunit of the K_ATP_ channel, and displace glibenclamide [47].

### 4.8. Cassia alata

*Cassia alata* (Caesalpiniaceae) is a shrub that is mostly utilized in traditional medicine in tropical nations such as Malaysia, Brazil, and Indonesia. The principal pharmacological actions listed in the literature for *Cassia alata* are the anti-inflammatory, antidiabetic, antifungal, and antibacterial effects [48,49,50].

Astragalin is one of the main flavonoids found in *C. alata* leaves. Rey et al. measured the glucose level and secretion of insulin in fasting Wistar rats after the oral administration of astragalin. The results of their study showed that astragalin at a concentration of 10 mg/kg significantly enhanced insulin secretion and lowered glycemia within 15–180 min of application. In isolated pancreatic cells, glibenclamide and diazoxide were applied to test the possibility that astragalin caused an increase in the Ca^2+^ influx that was mediated by K_ATP_ channels. The results showed that the Ca^2+^ influx increased 2.3 times more than the control when astragalin was applied in the presence of glibenclamide. On the other hand, diazoxide alone considerably reduced the influx of Ca^2+^. Furthermore, the stimulatory action of astragalin was reduced by almost 50% when diazoxide was present. These findings suggest that astragalin may act as an insulin secretagogue and support glucose homeostasis through a mechanism combining K_ATP_, L-type Ca^2+^ channels, and the sarcoendoplasmic reticulum Ca^2+^ transport ATPase (SERCA) that allow astragalin to induce Ca^2+^ influx in pancreatic cells [51].

### 4.9. Momordica charantia

*Momordica charantia*, also known as bitter melon, is a member of the Cucurbitaceae family and is mainly grown in Asia, Africa, and South America [52]. The plant’s medicinal effects and nutritive values have been well-known for decades. The plant is used in folk medicine around the world to treat a variety of pathologies, particularly diabetes, but also inflammation and cancer, due to the presence of several biologically active compounds [53]. It has been well established that *M. charantia* extract helps type 2 diabetic patients by reducing blood sugar levels.

Using different diabetic animal models, the bitter melon’s hypoglycaemic impact has been demonstrated [54,55]. In alloxan-induced diabetic rats, blood glucose tolerance was considerably improved with the chronic oral administration of *M. charantia* fruit juice [56]. In male ddY diabetic mice, the main pure cucurbutanoid compounds in *M. charantia* were shown to exhibit hypoglycaemic effects. The effects on blood glucose were nonetheless considerable, even if they were less pronounced than those of glibenclamide at the same dose [57].

Recently, the effect of bitter melon fruit on insulin secretion from pancreatic β-cells and their underlying mechanism was studied by Shimada et al. The results of this study showed that the fruit extract of bitter melon acutely lowered blood glucose levels in healthy and in STZ-induced diabetic animals. Furthermore, the extract enhanced insulin release from β-cells independent of the dose of glucose. The enhancement of insulin secretion by the fruit extract appeared to be caused by an observed increase in ATP generation in β-cells. Since increased ATP concentration in the β-cell results in the closure of K_ATP_ channels, it can be concluded that the effect of the bitter melon extract is directly linked to the inhibition of K_ATP_ channels. It is suggested that the hydrophobic components of bitter melon fruit may stimulate glucose metabolism in β-cells by upregulating the enzymes responsible for glucose metabolism and ATP synthesis in the mitochondria [58]. Supporting this finding, earlier studies have also reported that *M. charantia* extracts impact the enzymes involved in the glycolytic cascade, in addition to increasing insulin secretion and decreasing insulin resistance [59].

### 4.10. Zingiber officinale

*Zingiber officinale* (ginger) is an herbaceous plant belonging to the Zingiberaceae family. The plant is frequently used as a flavoring food. Ginger is also one of the most well-known therapeutic plants in traditional medicine for its antioxidant, anti-inflammatory, antinausea, anti-emetic, and anticancer effects [60]. Accumulated evidence has shown that ginger and its constituents gingerol, shogaol, and paradol have hypoglycemic effects by increasing insulin sensitivity and lowering the chance of developing DM. Zhu et al. showed that 6-shogaol and 6-gingerol suppressed the synthesis of AGEs by capturing methylglyoxal (MGO), the precursor of AGEs, in an in vitro experiment [61].

In high-fat diet-fed mice, 6-gingerol [62] and 6-paradol [63] decreased plasma glucose and insulin levels. In Lepr^db/db^ type 2 diabetic mice, the oral administration of 6-gingerol (200 mg/kg) for 4 weeks enhanced the cell membrane expression of glucose transporter type 4 (GLUT4) and activated glycogen synthase 1, which promoted glucose uptake in skeletal muscles [64]. Mahluji et al. showed that consumption of ginger by individuals with type 2 diabetes resulted in lower levels of insulin, low-density lipoprotein cholesterol, and triglycerides, a lower homeostasis model assessment index, and a higher quantitative insulin sensitivity check index. Therefore, it was suggested that ginger could be a helpful treatment to lessen DM-associated complications [65].

In comparison to either fresh or dried ginger, the steaming process has been proven to improve the efficacy of the ginger. According to the findings by Cheng et al., steamed ginger (120 °C, 4 h) had an antiproliferative effect that was approximately two times stronger than that of dry and fresh ginger [66]. A recent study revealed that steamed ginger contains more than 1-Dehydro-6-gingerdione and may increase the secretion of insulin through the inhibition of K_ATP_ channels in pancreatic cells. Using zebrafish as an animal model, the steamed ginger extract showed a higher efficacy in the recovery of alloxan-induced damage in pancreatic islets. Interestingly, the co-treatment of pancreatic islets with diazoxide in the presence of a steamed ginger extract and 1-Dehydro-6-gingerdione, resulted in considerably smaller pancreatic islets compared to pancreatic islets treated with a steamed ginger extract or 1-Dehydro-6-gingerdione alone. According to these findings, it was suggested that steamed ginger extract and 1-Dehydro-6-gingerdione increase insulin secretion by blocking K_ATP_ channels in pancreatic cells [67].

### 4.11. Kalanchoe pinnata

*Kalanchoe pinnata* (Crassulaceae) is a traditional medicinal plant that has been reported for its immunomodulatory [68], hepatoprotective [69], hypoglycemic [70], and anticancer [71] effects. Patil et al. assessed the antihyperglycemic and insulin secretagogue capabilities of *K. pinnata* in STZ-treated rats. Similar to glibenclamide, the dichloromethane component of the plant displayed a glucose-independent insulin secretagogue effect. Fasting blood glucose levels were dramatically lowered in STZ-induced diabetic rats after treatment with 10 mg/kg body weight of dichloromethane fraction. In addition, lipid profile and insulin levels were also normal. Moreover, glycated hemoglobin decreased to 8.4% from 12.9% in the diabetic controls. In vitro experiments showed that the insulin secretagogue activity of the dichloromethane fraction was dose-dependent and abolished by diazoxide. Therefore, the insulin secretagogue effect could be attributed to the closure of K_ATP_ channels [70]. Similarly, *Kalanchoe crenata*, another species of *Kalanchoe*, was also demonstrated to have antihyperglycemic effects in prior research. The modification of insulin sensitivity was hypothesized to be the cause of its antihyperglycemic action [4].

### 4.12. Heritiera fomes

*Heritiera fomes* is an evergreen tree belonging to the Sterculiaceae family which grows abundantly in Sundarbans. *H. fomes* has been extensively used in the treatment of various ailments, such as diabetes and cardiac and hepatic disorders [72]. Although *H. fomes* has remarkable therapeutic potential, there is little information on its mechanism of action or the composition of the active constituents that could be in charge of such favorable effects. Nonetheless, it is known that *H. fomes* contains a variety of potentially significant phytochemicals such as polyphenols, tannins, and carotenoids [72,73].

A recent study conducted by Ansari et al. tested the effect of hot water extract of *H. fomes* on insulin release from BRIN BD11 cells and isolated mouse islets, as well as on glucose homeostasis in high-fat-fed rats [73]. The oral administration of hot water extract to high-fat-fed rats significantly improved glucose tolerance and plasma insulin responses. Basal and glucose-induced insulin production was concentration-dependently enhanced by the hot water extract of *H. fomes in* BRIN BD11 cells and isolated mouse islets. The presence of the K_ATP_ channel blocker tolbutamide or depolarization with KCl did not interfere with the insulin secretory effect of the hot water extract *H. fomes*. Nevertheless, the effect was reduced by the K_ATP_ channel opener diazoxide, suggesting that the cellular actions of the hot water extract of *H. fomes* may entail the closing of K_ATP_ channels in addition to K_ATP_ channel-independent actions. In agreement with this finding, the L-type voltage-dependent Ca^2+^ channel blocker verapamil reduced the activity of the hot water extract of *H. fomes*, indicating that Ca^2+^ channels play a role in insulin secretion. This finding was further supported by the membrane depolarizing effect of the hot water extract and the resulting increase in intracellular Ca^2+^ in BRIN-BD11 cells. Phytochemical analysis revealed the presence of quercetin as the main active constituent of the water extract of *H. fomes* [73].

### 4.13. Enicostemma littorale

*Enicostemma littorale* is a member of the Gentianaceae family. According to previous research, the plant possesses anti-inflammatory [74], anti-cancer [75], and hypoglycemic effects. The long-term treatment (30 days) of diabetic rats with the aqueous extract of *E. littorale* has shown a significant reduction in blood glucose and glycosylated hemoglobin levels [76]. In alloxan-induced diabetic rats, a single dosage of the aqueous extract of *E. littorale* has demonstrated a substantial rise in blood insulin levels [77]. Using rat pancreatic islets, the insulinotropic effect of the aqueous extract of *E. littorale* was also confirmed. The extract potentiated glucose-induced insulin release from isolated rat pancreatic islets and was partially able to reverse the effect of diazoxide. Furthermore, the glucose-induced insulin release was unaffected by the presence of the Ca^2+^ channel blocker nimodipine. The results of this study indicated that the aqueous extract of *E. littorale* may possess a glucose-lowering effect by potentiating the release of insulin when blood sugar levels are elevated. This effect was dependent on K_ATP_ channels and did not require Ca^2+^ influx [77].

### 4.14. Tabernanthe iboga

*Tabernanthe iboga* belongs to the Apocynaceae family and is widely grown in the tropical forest of Central Africa. Root bark preparations have long been used in traditional medicine in Central and West African regions for the management of type 2 diabetes [78]. Despite this, scientific evidence on the antidiabetic effects of *T. iboga* is very limited.

Souza et al. showed that the aqueous extract of *T. iboga* had sulfonylureas-like action on insulin release [79]. In isolated pancreatic rat islets, the aqueous extract significantly increased the secretion of insulin in response to mM glucose stimulatory concentrations by closing K_ATP_ channels and increasing the influx of Ca^2+^ [79]. Similarly, Bading-Taïka et al. also showed that *T. iboga* aqueous extract (50 to 200 mg/kg p.o.) had a hypoglycemic impact on healthy rats [78]. This effect was delayed and only manifested after 3 h, which may be indicative of the intracellular action of the compound as previously demonstrated in isolated pancreatic β-cells by Souza et al. [79]. Additionally, *T. iboga* aqueous extract showed antihyperglycemic effects on non-fasted and fasted blood glucose in fructose-fed STZ diabetic rats [78].

### 4.15. Teucrium polium

*Teucrium polium* belongs to the family Lamiaceae and is mostly found in the Mediterranean and western Irano-Turanian regions [80]. This medicinal plant, also known as calpoureh, is traditionally used as a spice, a hypoglycemic agent, or a herbal tea by herbalists. Many therapeutic properties of this plant have been documented, including antioxidant, anti-inflammatory, hypolipidemic, and hypoglycemic effects [80,81,82]. Mirghazanfari et al. investigated the impact of *T. polium* extracts on insulin production from an isolated rat pancreas [83]. Interestingly, only the methanolic extract, but not the aqueous extract of *T. polium,* caused a significant increase in insulin release. According to the study findings, both diazoxide and verapamil decreased the insulinotropic activity of the *T. polium* extract, suggesting that the mechanism of insulin secretion was mediated by Ca^2+^ channel activation, K_ATP_ channel inhibition, or both. Moreover, it was determined that only the methanolic extract of *T. polium* contained the chemical apigenin, which is responsible for the insulinotropic activity of *T. polium* [83].

### 4.16. Nelumbo nucifera

*Nelumbo nucifera* (Lotus) is a perennial aquatic plant that belongs to the family *Nelumbonaceae* [84]. All plant parts have been widely used in traditional medicine for many medicinal purposes. For example, the herbal tea from dry leaves of *N. nucifera* is used to lose weight and decrease body fat index, while herbal tea from stamens is commonly used to improve the circulatory system and to decrease blood glucose and lipid levels [85]. Nguyen et al. found that the alkaloid nuciferine extracted from *N. nucifera* stimulated insulin secretion in both isolated islets and INS-1E cells. Nuciferine acted primarily by closing K_ATP_ channels. The effect of nuciferine was eliminated by diazoxide and nimodipine and decreased by protein kinase A and protein kinase C inhibition, indicating that the alkaloid also acted through K_ATP_ channel-independent pathways. Interestingly, nuciferine had a weaker affinity for binding to the sulfonylurea receptor, a stronger effect on insulin secretion, and less cytotoxicity compared to glibenclamide [86].

### 4.17. Gynostemma pentaphyllum

*Gynostemma pentaphyllum*, also known as jiaogulan, is a perennial climbing vine that belongs to the family Cucurbitaceae and is found across many Asian countries. The key phytochemicals include saponins and sterols [87]. Many studies have established the pharmacological benefits of *G. pentaphyllum*. The plant’s great potential for scavenging free radicals, in addition to its anti-inflammatory, hypoglycemic, and anticancer properties, have all been previously noted [88,89,90,91]. In both randomly selected type 2 diabetic individuals and diabetic animal models, the hypoglycemic effects of *G. pentaphyllum* were demonstrated. The administration of *G. pentaphyllum* tea to diabetic patients enhanced their ability to tolerate glucose by increasing their insulin sensitivity [91]. Similarly, in the Goto-Kakizaki rat, *G. pentaphyllum* extract lowered hepatic glucose production [92]. Moreover, *G. pentaphyllum* saponins were found to cause hypoglycemia and hypolipidemia in the diabetic rat model [93]. A more recent study examined the mechanism of antidiabetic action on the aqueous extract of *G. pentaphyllum* in Goto-Kakizaki diabetic rats [94]. According to the study findings, the plant’s extract enhanced glucose tolerance, raised plasma insulin levels and boosted the release of insulin from isolated rat islets. Moreover, extract-induced insulin release was partially mediated via K_ATP_ channels, L-type Ca^2+^ channels, and protein kinase: a system and pertussis toxin-sensitive G_e_-protein at high glucose levels [94]. The insulinotropic characteristics of *G. pentaphyllum* were attributed to the presence of phanoside, an active gypenoside molecule, which has been reported to possess a strong insulin-releasing action [95].

### 4.18. Swietenia humilis

*Swietenia humilis*, commonly known as zopilote, belongs to the family Meliaceae. The plant is among the species widely used to alleviate diabetes and dyslipidemia [96,97,98] Tetranortriterpenoids of the mexicanolide class were recently isolated from the seeds of *S. humilis*. Ovalle-Magallanes et al. tested the effects of three mexicanoloides in vivo (using STZ-treated mice) and in vitro (using INSE1, H4IIE, and C2C12 cells) to determine the mechanisms of antihyperglycemic activity on *S. humilis* [97]. The results of this study demonstrated that the mexicanolide 2-hydroxy-destigloyl-6-deoxyswietenine acetates modulate glucose homeostasis by interacting with a variety of pharmacological targets including the pancreas (K_ATP_ channels), liver (glucose-6-phosphatase) and skeletal muscle (mitochondria and possibly glucose transporters). However, the fact that the compound retained a minimal antihyperglycemic effect in the presence of diazoxide indicated that K_ATP_ channels were only partially involved in the antihyperglycemic action of this compound.

Table 1 summarizes the effects of different antidiabetic medicinal plants and their active constituents on K_ATP_ channels.

**Figure 2:**
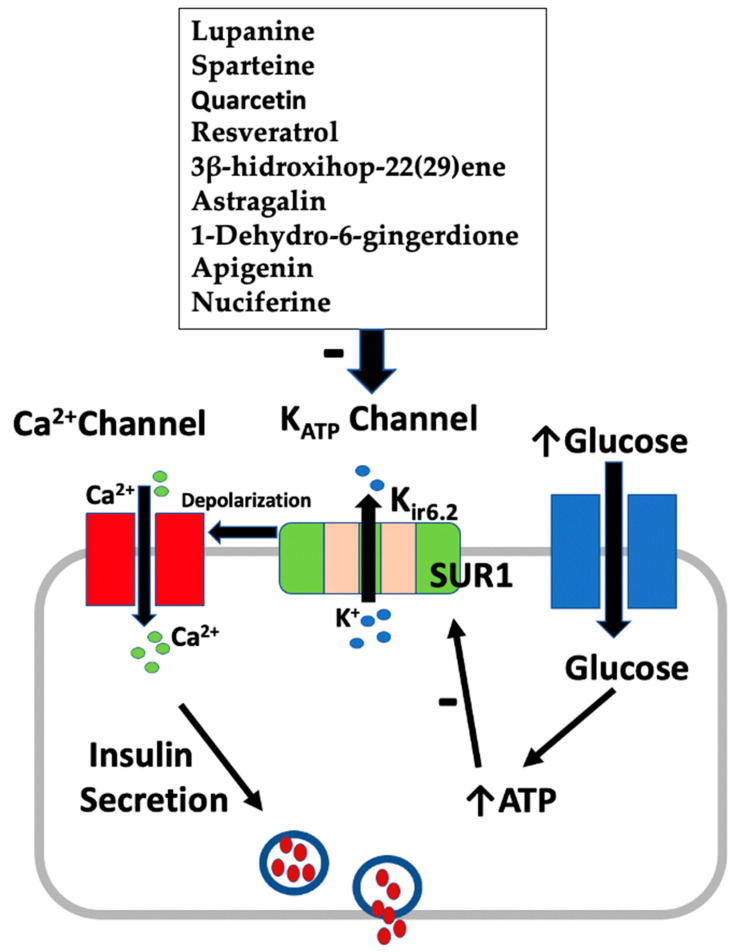
Effect of active plant constituents on insulin secretion from β-cells.

Figure 2 summarizes the mechanism of insulin secretion from pancreatic β-cells by active plant constituents.

## 5. Other Plants with Potential Modulatory Effect on K_ATP_ Channels

### 5.1. Berberis aristata

*Berberis aristata* belongs to the family Berberidaceae. The plant is native to the northern Himalayan region and is commonly referred to as Citra or Daruhaldi. The extract of the roots of *B. aristata* has potent antioxidant and anti-hyperglycemic effects [99,100]. Berberine is the main antidiabetic substance extracted from this plant. Berberine is known to act mainly by mimicking the action of insulin, reducing insulin resistance, and upregulating the expression of insulin receptors [99]. In a 6-month randomized controlled study in type 1 diabetic subjects, the *Berberis aristata*/*Silybum marianum* combination reduced the body’s use of insulin during insulin treatment. Hb1Ac levels were decreased in comparison to the baseline. Furthermore, fasting and postprandial levels of plasma glucose were also decreased in comparison to the baseline and placebo [101]. Similarly, a year-long placebo-controlled trial found that this combination, which intended to increase the poor bioavailability of berberine when taken orally, had significant antidiabetic benefits in type 2 diabetic subjects [102].

The effect of berberine on the K_ATP_ channel was demonstrated in an earlier study. In a whole-cell voltage clamp experiment, berberine inhibited the cromakalim-induced outward K^+^ currents in a concentration-dependent manner and decreased open channel probability. Such inhibition was completely blocked by glibenclamide, indicating that the observed effect of berberine is due to the inhibition of the K_ATP_ channel. However, the effect of this compound was tested on cardiac K_ATP_ only, and currently, no studies have shown the link between berbeine and pancreatic K_ATP_ [103].

### 5.2. Psacalium decompositum

*Psacalium decompositum* (Asteraceae), sometimes referred to as “matarique,” is a prominent remedy in Mexican folk medicine for the treatment of neuralgia, hepatic and renal colic, rheumatic illnesses, discomfort, and ulcers [104]. Previous research looked at the mechanism underlying the hypoglycemic effects of aqueous root and rhizome decoctions of *P. decompositum.* The hypoglycemic effects of the three sesquiterpenoids, cacalol, cacalol acetate, and cacalone epimer mixture, were investigated. Similar to glibenclamide, all the tested compounds inhibited K_ATP_ channels in aortic smooth muscle rings. The sesquiterpenoids may have a stronger affinity for the SUR2 subunit of K_ATP_ channels in aortic smooth muscle than the SUR1 subunit in pancreatic β-cells since they were less successful than glibenclamide in decreasing plasma glucose levels [105].

### 5.3. Annona cherimola

*Annona cherimola* is a perennial plant that belongs to the Annonaceae family. The plant’s fruit is known as “annona” or “cherimoya” [96]. The leaves were used in traditional medicine as a remedy to treat gastrointestinal disorders [106]. Studies have also demonstrated that the leaves possess antidepressants [107] and pro-apoptotic properties [108]. Interestingly, the administration of the tea infusion extract of leaves from *A. cherimola* improved blood glucose and glycated hemoglobin levels in mice [109]. Recently, Valdes et al. demonstrated that the ethanolic extract of *A. cherimola* and the flavonoid rutin administered alone and in combination with oral antidiabetic drugs caused a significant reduction in hyperglycemia, glycated hemoglobin, and hyperlipidemia in STZ-treated mice [110]. Although many phytochemicals with reported hypoglycemic and insulin-secreting effects (such as flavonoids, alkaloids, and sesquiterpenes) have been isolated from *A. cherimola*, there are currently no studies on the effect of this plant extract on K_ATP_ channels.

### 5.4. Bougainvillea spectabilis

*Bougainvillea spectabilis*, which is commonly known as Bougainvillae, belongs to the Nyctaginaceae family. It is a woody and thorny vine cultivated in the tropical and subtropical regions of India. The plant leaves are rich in flavonoids, quinones, phenols, sterols, triterpenoids, glycosides, and tannins [111]. The extract of its leaves contains pinitol, which has been shown to possess insulin-like activity [112]. The daily administration of the aqueous extract of *B. spectabilis* leaves at a dose of 100 mg/kg body weight for 28 days resulted in a significant reduction in hyperglycemia in STZ-pretreated rats [111]. A recent study has shown that the methanol extract of *B. spectabilis* leaves has an antinociceptive effect, possibly through the modulation of K_ATP_ channels. Therefore, further investigation is needed to test the effect of *B. spectabilis* extracts on K_ATP_ channels in the pancreas.

### 5.5. Lycium barbarum

*Lycium barbarum* belongs to the family Solanaceae. The plant is well known as “Ausaj” and “Box Thorn” and has been used in traditional medicine to treat several ailments such as cancer and hepatitis and to reduce blood sugar [113].

Recently, Hager et al. identified many distinct plant extracts and some of their bioactive constituents as modulators of insulin production in β-cells. In this study, *L. barbarum* extracts were identified among the highest plant extracts with stimulatory activity on insulin secretion [114]. The effect of the polysaccharide galactomannan from *L. barbarum* fruit was studied in alloxan-induced diabetic rats. The maximal effect of the polysaccharide extract (500 mg/kg) administered with glibenclamide occurred at 6 h and faded after 24 h. A chronic application, where glibenclamide and polysaccharide galactomannan was repeatedly administered once daily for 21 days, further resulted in a substantial decrease in blood glucose levels compared to the diabetic control group. Considering the similar hypoglycemic effects determined by the polysaccharide galactomannan with respect to glibenclamide, it was proposed that their mechanism of action was the same (via the inhibition of K_ATP_ channels) [113]. However, further data are needed to confirm the K_ATP_ channels-dependent hypoglycemic effect of *L. barbarum.*

## 6. Conclusions

Medicinal plants have been extensively used in folk medicine to treat DM. K_ATP_ channels are metabolic detectors that are required for regular insulin production. Although a lot of information is available on the antidiabetic effects of medicinal plants and their active constituents, very limited studies discuss their direct action on pancreatic K_ATP_. The available data affirms the need for further research into the interaction of medicinal plants with the K_ATP_ channel. Future studies are anticipated to use natural substances as lead structures for the manufacture of therapeutic drugs that target K_ATP_ channels.

## Figures and Tables

**Table 1:** Effect of antidiabetic medicinal plants and their active constituents on insulin secretion through the inhibition of pancreatic K_ATP_ channels.

Plant Source	Constituent	Study Type	Subject	Dose	Effect	Reference
*Lupinus mutabilis*	Lupanine	In vitro	β-cell line (INS-1E)	0.5 mmol/L	Inhibition of K_ATP_ channels	[22]
		In vivo	STZ-pretreated rat	0.5 mmol/L	Stimulation of insulin secretion	[22]
	Sparteine	In vitro	β-cell line (HIT-T15)	2–20 μM	Inhibition of K_ATP_ channels	[24]
		In vitro	Mouse β-cell	0.02–0.5 mM	Potentiation of glucose effect, membrane depolarization, and increase insulin secretion by reducing K^+^ permeability	[23]
*Portulaca oleracea*	3β-hidroxihop-22(29)ene	In vitro	Rat pancreatic β-cell	100 nM	Increase in glucose uptake, insulin vesicles translocation to the plasma membrane and increase in insulin secretion	[41]
	Aqueous extract	In vitro	INS-1E β-cells	10–200 µg/mL	Increase in insulin secretion and intracellular Ca^2+^ and inhibition of K_ATP_ channels	[40]
*Belamcanda chinensis*	Aqueous extract	In vivo	STZ-induced diabetic rat	800–1600 mg/Kg	Suppression of hyperglycemia, Enhancement of insulin secretion via K_ATP_ channel	[27]
*Hyphaene thebaica*	Aqueous extract	In vivo	Alloxan induced diabetic rats	20 mg/Kg	Improvement in glucose and insulin tolerance, decrease in glycosylated hemoglobin levels	[29,31]
		In vitro	β-cells of STZ-induced diabetic rat		Decrease in β-cell necrosis and inhibition of K_ATP_ channels	[31]
*Eucalyptus citriodora*	Quercetin	In vitro	Rat INS-1 β-cells	50 μM	Depolarization of β-cell membrane, increase in intracellular Ca^2+^ concentration, and decrease in K_ATP_ current	[36]
*Leonurus sibiricus*	Aqueous and Methanolic extracts	In vitro	INS-1E β-cells	500 mg/L	Increase in insulin secretion, increase in intracellular Ca^2+^, and inhibition of K_ATP_ channels	[38]
*Cichorium intybus*	Resveratrol	In vitro	Mouse β-cell line	3–100 μmol/L	Inhibition of K_ATP_ current and stimulation of insulin secretion by causing membrane depolarization	[44,45,46,47]
*Cassia alata*	Astragalin	In vivo	Wistar rats	10 mg/kg	Enhancement of insulin secretion and hypoglycemic effect	[51]
		In vitro	Rat pancreatic β-cells		Stimulation of L-type Ca^2+^ channels and Ca^2+^ influx, and inhibition of K_ATP_	[51]
*Zingiber officinale*	1-Dehydro-6-gingerdione	In vitro	STZ-treated mouse	100 mg/kg (4 weeks)	Lowering of hyperglycemia and stimulation of insulin secretion by inhibition of K_ATP_	[67]
*Kalanchoe Pinnata*	Dichloromethane fraction	In vitro	STZ-treated rat	10 mg/kg body weight	K_ATP_-dependent insulin secretagogue effect	[70]
*Heritiera fomes*	Hot water extract	In vivo	High-fat-fed rat	250 mg/5 mL/kg body weight	Improvement in glucose tolerance and plasma insulin responses	[73]
		In vitro	BRIN-BD11 cells Isolated mouse islets	≥1.6 μg/mL ≥25 μg/mL	Depolarization of β-cell membrane, increase in intracellular Ca^2+^ concentration, decrease in K_ATP_ current and increase in insulin secretion Increase in insulin secretion	
*Enicostemma littorale*	Aqueous extract	In vivo	Alloxan-induced diabetic rats	15 g dry plant equivalent extract per kg	Potentiation of insulin release via K_ATP_ dependent pathway	[77]
	Aqueous extract	In vitro	Rat pancreatic islets	25 μg	Potentiation of glucose-induced insulin release through K_ATP_ channel dependent pathway	[77]
*Tabernanthe iboga*	Aqueous extract	In vitro	Rat pancreatic islets	1 mg/mL	Increased insulin secretion by closing K_ATP_ channels and increasing the influx of Ca^2+^	[79]
*Teucrium polium*	Methanolic extract	In situ	Isolated perfused rat pancreas	1 mg/mL	Potentiation of glucose-induced insulin release through K_ATP_ and Ca^2+^ channels dependent pathway	[83]
*Nelumbo nucifera*	Nuciferine	In vitro	Isolated islets and INS-1E cells	10 μM	Stimulation of insulin secretion, primarily through the blockage of K_ATP_ channels	[86]
*Gynostemma Pentaphyllum*	Aqueous extract	In vitro	Rat pancreatic islets	0.3 g/kg body weight daily	Potentiation of insulin secretion mediated by K_ATP_ channels, L-type Ca^2+^ channels, and protein kinase A system	[93,94]
*Swietenia humilis*	2-hydroxy-destigloyl-6-deoxyswietenine acetate	In vitro	INS-1E cells	2 mg/kg	K_ATP_ channels-dependent and K_ATP_ channels-independent hypoglycemic effects	[97]

## Data Availability

Data sharing not applicable.
